# Functional neuroimaging and chorea: a systematic review

**DOI:** 10.1186/s40734-017-0056-0

**Published:** 2017-06-21

**Authors:** Debra J. Ehrlich, Ruth H. Walker

**Affiliations:** 1 Department of Neurology, Icahn School of Medicine at Mount Sinai, 5 East 98th Street, 1st Floor, Box 1637, New York, NY 10029 USA; 2 Department of Neurology, James J Peters Veterans Affairs Medical Center, 130 West Kingsbridge Road, Bronx, NY 10468 USA

**Keywords:** Chorea, Functional imaging, Positron emission tomography, Functional MRI, Single-photon emission tomography, Huntington, Acanthocytosis, Sydenham

## Abstract

Chorea is a hyperkinetic movement disorder consisting of involuntary irregular, flowing movements of the trunk, neck or face. Although Huntington’s disease is the most common cause of chorea in adults, chorea can also result from many other neurodegenerative, metabolic, and autoimmune conditions. While the pathophysiology of these different conditions is quite variable, recent advances in functional imaging have enabled the development of new methods for analysis of brain activity and neuronal dysfunction. In this paper we review the growing body of functional imaging data that has been performed in chorea syndromes and identify particular trends, which can be used to better understand the underlying network changes within the basal ganglia. While it can be challenging to identify whether changes are primary, secondary, or compensatory, identification of these trends can ultimately be useful in diagnostic testing and treatment in many of the conditions that cause chorea.

## Introduction

Chorea is a hyperkinetic movement disorder consisting of involuntary irregular, flowing movements of the limbs, trunk, neck or face. While Huntington’s Disease (HD) is the most common cause of chorea in adults [1], chorea can be a prominent symptom in a variety of neurologic diseases, including neurodegenerative, metabolic, and autoimmune conditions. While the pathophysiology of chorea is most commonly linked to dysfunction of inhibitory pathways within the basal ganglia, the neuronal basis underlying the generation of chorea appears to be quite heterogeneous. For many years, neuroimaging techniques focused mainly on structural changes, however, recent advances in functional imaging have enabled an in vivo analysis of neuronal dysfunction and brain activity.

HD is a progressive neurodegenerative condition for which disease-modifying therapies are currently lacking. Therefore, treatment of HD is symptomatic only and mainly aimed at treating the chorea and psychiatric symptoms. Treatment of chorea in HD is usually limited to dopamine-depleting agents, dopamine antagonists, and anti-glutamatergic agents. These medication classes are also frequently used to treat chorea from other etiologies, including neurodegenerative, metabolic, and autoimmune. The efficacy of common therapies in reducing chorea regardless of etiology suggests a common mechanism underlying the pathogenesis of chorea. Functional imaging can be useful in elucidating underlying commonalities amongst various conditions that cause chorea.

The aim of this review is to collate a variety of results from functional imaging studies in patients with chorea from a variety of etiologies to identify common or distinct patterns. We propose that functional imaging studies of conditions with chorea as a symptom could reveal particular trends that could be useful in the diagnosis and treatment of these conditions and in understanding the underlying basal ganglia network changes.

## Methods

A literature search was conducted using PubMed (February-April 2016) utilizing various combinations of the following key words: “positron emission tomography,” “PET,” functional magnetic resonance imaging,” “fMRI,” “resting state fMRI,” “single photon emission computed tomography,” “SPECT,” “Huntington,” “chorea,” “acanthocytosis,” “dentatorubropallidoluysian atrophy,” “benign hereditary chorea,” “spinocerebellar ataxia,” “Sydenham,” and “systemic lupus erythematosus.” Additional papers were found using references from articles yielded by the PubMed search. Animal studies were excluded and only papers written in English and published between January 1982 and April 2016 were reviewed. A total of 2174 papers were obtained from the literature review and each article was screened for relevancy to the topic. Only articles cited in this paper are included in the references section.

## Review

### Positron emission tomography

#### Positron emission tomography in HD

Positron emission tomography (PET) is a functional imaging technique which involves the injection of a radiolabeled ligand that either binds to specific structures of interest, such as neurotransmitter receptors, or is incorporated into the body’s tissues in other ways, such as ^18^F-fludeoxyglucose (FDG). PET imaging can be informative in cases of chorea arising from various etiologies to learn more about pathogenesis, disease course, and basal ganglia functions.

PET scans using the radionuclide FDG are employed to demonstrate changes in cerebral glucose metabolism. Several FDG-PET studies in symptomatic HD patients have demonstrated significantly reduced glucose metabolism in the caudate nucleus and putamen [2–5], correlating with the severity of neurologic signs and symptoms [3] (see Table 1). Decreased glucose metabolism has also been demonstrated in pre-manifest HD mutation carriers in the putamen, caudate, and globus pallidus [6]. Longitudinal FDG-PET scans in pre-manifest mutation carriers revealed a progressive decline in glucose metabolism in the caudate, putamen, thalamus, insula, and posterior cingulate gyrus, and prefrontal and occipital cortex, while increases in glucose metabolism were found in the cerebellum, pons, hippocampus, and orbitofrontal cortex [7]. Additionally, decreases in putaminal [6] and caudate [8] metabolism have been correlated with time to phenoconversion. These findings were independent of volume loss and suggest that there are progressive changes in metabolic network connectivity within both the basal ganglia and other brain regions, which may precede the clinical onset by many years. As these patients did not yet have any neurologic or psychiatric manifestations of HD, the findings cannot be secondary to their symptoms.

**Table 1:** PET and SPECT in Huntington’s disease

Authors/Year	Imaging modality	Radioligand or tracer	Target/Purpose of tracer	Number of subjects	Main findings
Brain metabolism					
1982 Kuhl et al. [4]	PET	[^18^F]FDG	Regional glucose metabolism	13 HD, 15 offspring of HD patients, 40 HC	C/Pu hypometabolism began shortly after symptom onset and prior to tissue loss/atrophy
1986 Young et al. [3]	PET	[^18^F]FDG	Regional glucose metabolism	15 HD, 14 HC	C/Pu hypometabolism, degree of which correlated with stage on Shoulson and Fahn scale
2001 Feigin et al. [2]	PET	[^18^F]FDG	Regional glucose metabolism	18 pre-HD, 13 early HD, 8 gene negative relatives	C/Pu hypometabolism in pre-HD and early HD
2012 Ciarmiello et al. [8]	PET	[^18^F]FDG	Regional glucose metabolism	43 pre-HD	Reduction in C metabolism can predict time to phenoconversion
2014 Herben-Dekker et al. [6]	PET	[^18^F]FDG	Regional glucose metabolism	22 pre-HD, 11 HC	C/Pu, GP hypometabolism; on 2-year follow-up all phenoconverted patients exhibited Pu hypometabolism; Pu metabolism remained normal in asymptomatic gene carriers
Cerebral perfusion					
2002 Reynolds et al. [52]	SPECT	^99m^Tc exametazime, ^99m^Tc Bicisate	Regional cerebral perfusion	34 HD, 12 pre-HD	Most HD patients and 5/12 pre-HD exhibited C hypoperfusion, 7/12 pre-HD showed normal C perfusion
Dopaminergic function					
1999 Andrews et al. [9]	PET	[^11^C]SCH 23390, [^11^C]raclopride	D1 and D2	9 pre-HD, 10 HD, 11 at risk HD (6 gene negative, 5 not tested)	Progressive reduction in D1 and D2 receptor binding in C/Pu in pre-HD
1999 Leslie et al. [51]	SPECT	[^123^I]IBZM	D2	21 HD (varying stages), 11 HC	Reduced in striatum in moderate-severe stages of HD; normal in pre-HD and early symptomatic HD
2003 Pavese et al. [12]	PET	[^11^C]raclopride	D2	12 HD	Progressive reduction in C/Pu D2 receptor binding (not associated with UHDRS motor scores), progressive reduction in D2 binding in frontal cortex and temporal cortex
2009 Van Oostrom et al. [10]	PET	[^11^C]raclopride	D2	27 pre-HD, 14 HC	At baseline and 2-year follow-up reduced D2 binding in Pu in pre-HD, weakly correlating with probability of symptom onset within next 5 years
2010 Gamez et al. [47]	SPECT	^123^-I-FP-CIT	Presynaptic dopamine transporters	12 HD	Reduced in C/Pu in the majority
2011 Esmaeilzadeh et al. [13]	PET	[^11^C]FLB 457	D2	9 HD, 9 HC	Decreased D2 binding in Pu correlated with maximal chorea score (UHDRS item 12) and scores on cognitive testing; reduced D2 binding in C correlated with modified motor score (UHDRS items 4–10, 13–15)
2013 Hwang et al. [50]	SPECT	[^99^Tc]TRODAT-1, [^123^I]IBZM	Dopamine transporter, D2	3 HD (related family members), 1 mutation negative member of HD family, 7 HC	Reduced D2 binding in striatum of HD patients, striatal dopamine transporter binding reduced only in the most symptomatic HD patient
2013 Kiferle et al. [48]	SPECT	^123^-I-FP-CIT	Presynaptic dopamine transporters	12 HD, 12 HC	Reduced in C/Pu in HD
2014 Gamez et al. [49]	SPECT	^123^-I-FP-CIT	Presynaptic dopamine transporters	4 HD	Progressive reduction in C/Pu on 2-year follow-up
PDE10					
2014 Russell et al. [17]	PET	[^18^F]MNI-659	PDE 10	3 pre-HD, 8 HD, 9 HC	Progressive decrease in pre-HD and HD in C/Pu, GP strongly correlated with UHDRS motor subscores
2016 Russell et al. [18]	PET	[^18^F]MNI-659	PDE 10	2 pre-HD, 6 HD, 11 HC	Progressive decrease in C/Pu, GP correlated with HD disease progression
Multiple tracer subtypes					
1996 Antonini et al. [5]	PET	[^18^F]FDG, [^11^C]raclopride	Regional glucose metabolism, D2	8 HD, 10 pre-HD, 9 gene negative members of HD families	C/Pu hypometabolism in HD/pre-HD, reduced [^11^C] raclopride binding in C/Pu in HD/pre-HD; both correlated with CAG repeat number
2007 Feigin et al. [11]	PET	[^18^F]FDG, [^11^C]raclopride	Regional glucose metabolism, D2	12 pre-HD	Elevated baseline thalamic metabolism in pre-HD with subsequent subnormal thalamic metabolism after symptom onset, progressive reduction in C/Pu D2 binding
2008 Politis et al. [14]	PET	[^11^C]raclopride, [^11^C]-PK	D2, marker of microglial activation	9 HD, 10 pre-HD, 9 HC in [^11^C]raclopride study and 10 HC for [^11^C]-PK	Reduced D2 binding and microglial activation in the hypothalamus in HD and pre-HD
2013 Tang [7]	PET	[^18^F]FDG, [^11^C]raclopride	Regional glucose metabolism, D2	47 pre-HD (longitudinal imaging performed in 21), 62 HC	Progressive reduction in glucose metabolism in C/Pu, thalamus, insula, and posterior cingulate gyrus, prefrontal cortex, occipital cortex. Progressive increase in glucose metabolism in cerebellum, pons, hippocampus, orbitofrontal cortex. Reduction in baseline D2 binding in C/Pu in pre-HD with subsequent linear decline in D2 binding in C/Pu binding which correlated with disease progression

Key studies using PET and SPECT imaging in HD and their main findings are summarized

*Abbreviations*: *C* caudate, *[* ^*11*^ *C]FLB 457* (S)-N-((1-ethyl-2-pyrrolidinyl)methyl)-5- bromo-2,3-dimethoxybenzamide, *[* ^*11*^ *C]-PK* [^11^C]-R-PK11195, *[* ^*11*^ *C]SCH 23390* (R)-(+)-8-Chloro-2,3,4,5-tetrahydro-3-[^11^C]methyl-5-phenyl-1H-3-benzazepin-7-ol, *D1* dopamine D1 receptor, *D2* dopamine D2 receptor, *FDG* fluorodeoxyglucose, *[* ^*18*^ *F]MNI-659* 92-(2-(3-(4-(2-[^18^F]fluoroethoxy)phenyl)- 7-methyl-4-oxo-3,4-dihydroquinazolin-2-yl)ethyl)-4- isopropoxyisoindoline-1,3-dione), *GP* Globus pallidus, *HC* healthy controls, *HD* Huntington’s Disease, *[* ^*123*^ *I]-FP-CIT* ^123^I-2B-carbomethoxy-3B-(4-iodophenyl)-N-(3-fluoro-propyl) nortropane), *[* ^*123*^ *I]IBZM* [^123^I]Iodobenzamide, *PDE10* Phosphodiesterase 10, *PET* Positron emission tomography, *pre-HD* asymptomatic patients with positive HD genetic testing, *Pu* putamen, *SPECT* Single Photon Emission Computed Tomography, *[* ^*99m*^ *Tc]TRODAT* (99m)Tc-[2[[2-[[[3-(4-chlorophenyl)-8-methyl-8-azabicyclo[3,2,1]-oct-2-yl]-methyl](2-mercaptoeythl)amino]ethyl]amino]ethane-thiolato(3-)N2,N2’, S2,S2]oxo-[1R-exo-exo)]), *UHDRS* Unified Huntington’s Disease Rating Scale

Another type of PET scan employs the use of the radioligands (R)-(+)-8-Chloro-2,3,4,5-tetrahydro-3-(9)methyl-5-phenyl-1H-3-benzazepin-7-ol ([^11^C]SCH 23390) and [^11^C]raclopride, selective dopamine D1 and D2 receptor antagonists respectively, to assess the degree of dopamine receptor binding. PET studies using these radioligands have demonstrated a significant loss of caudate and putamen D1 and D2 dopamine receptor binding in both HD patients and asymptomatic mutation carriers compared to controls [9]. Additionally, symptomatic HD patients and the majority of asymptomatic mutation carriers showed progressive loss in caudate and putamen D1 and D2 receptor binding with follow-up imaging [9]. In patients with pre-manifest HD, decreased [^11^C]raclopride binding in the putamen was seen at baseline and 2-year follow-up studies, and the degree of reduced binding showed a weak correlation with increased probability of symptomatic onset within the next 5 years [10]. Subsequent PET studies using [^11^C]raclopride have confirmed progressively reduced D2 receptor binding levels in the caudate and putamen [7, 11] as well as reduced D2 receptor binding in the amygdala, frontal cortex, and temporal cortex in symptomatic HD patients [12]. However, a later PET study using the radioligand (S)-N-((1-ethyl-2-pyrrolidinyl)methyl)-5- bromo-2,3-dimethoxybenzamide ([^11^C] FLB 457) with high-affinity for dopamine D2 receptors found no difference between extrastriatal (thalamus, temporal cortex, cerebellum) D2 receptor binding in HD patients compared to normal controls [13]. Reduced [^11^C]raclopride binding in the hypothalamus was demonstrated in both symptomatic and pre-manifest HD patients compared to controls [14]. Additionally, the decrease in D2 receptor binding in the striatum with [^11^C] FLB 457 PET correlated with scores on cognitive testing and severity of chorea [13]. These observations likely correlate with a progressive loss of striatal D1 and D2 dopamine binding with the degeneration of nigrostriatal and striatal medium spiny projection neurons as pre-HD progresses to symptomatic HD, and evidence suggests that this loss may correlate with development of both motor and cognitive symptoms in HD.

Phosphodiesterase 10 (PDE10) is a protein that is particularly prevalent in medium spiny neurons in the striatum, which is downregulated in mouse models of early HD [15] suggesting that it could be a useful biomarker for HD in humans. (2-(2-(3-(4-(2-[^18^F]fluoroethoxy)phenyl)- 7-methyl-4-oxo-3,4-dihydroquinazolin-2-yl)ethyl)-4- isopropoxyisoindoline-1,3-dione) ([^18^F]MNI-659) is a PET biomarker with specificity for PDE10 [16]. PET studies in early HD patients have demonstrated significantly reduced striatal [^18^F]MNI-659 compared to healthy controls [17, 18]. Additionally, in early HD patients, there was a significant further decline in [^18^F]MNI-659 uptake after one year compared to healthy controls [18]. These findings also suggest progressive neuronal cell loss in the striatum in HD patients.

#### PET in chorea of other etiologies

Similar to the findings in HD, FDG-PET imaging in patients with chorea-acanthocytosis (ChAc) has also demonstrated marked glucose hypometabolism in the caudate nucleus and putamen, albeit only in single cases or small series, due to the rarity of this disorder [19–24] (see Table 2). Asymmetrically decreased glucose uptake in the right compared to the left striatum was reported in monozygotic twins with ChAc [25]. Another case report using PET with ^15^O-labeled carbon dioxide also showed a severe reduction of regional oxygen metabolism in the putamen and caudate head, with a less profound reduction in the thalamus and the frontal lobe in ChAc [26].

**Table 2:** PET and SPECT in chorea of non-HD etiology

Etiology of chorea	Year/Authors	Imaging modality	Radioligand or tracer	Target/Purpose of tracer	Number of subjects	Main findings
ChAc	1989 Dubinsky et al. [21]	PET	[^18^F]FDG	Regional glucose metabolism	2 ChAc (brothers)	Decreased in C/Pu
	1991 Brooks et al. [22]	PET	[^18^F]FDG, [^11^C]raclopride, C^15^O_2_	Regional glucose metabolism, D2 binding, regional cerebral blood flow	[^18^F]FDG - 6 ChAc; [^11^C]raclopride with steady-state inhalation of C^15^O_2_ -30 HC, 16 levodopa responsive PD, 3 ChAc	Normal [^18^F]FDG uptake in C and anterior Pu, reduced [^18^F]FDG uptake in posterior Pu(similar to PD), reduced [^11^C]raclopride uptake in C > Pu, reduced regional blood flow to C/Pu
	1998 Tanaka et al. [26]	PET	^15^O labeled O_2_, ^15^O labeled CO_2_	Regional cerebral metabolic oxygen rate, regional cerebral blood flow	3 ChAc, 7 HC	Reduced regional cerebral blood flow and oxygen metabolism in C/Pu, bilateral frontal and left temporal regions; reduced cerebral blood flow in left parietal and bilateralthalamic regions
	2006 Muller-Vahl et al. [25]	PET, SPECT	[^18^F]FDG, ^123^-I-FP-CIT	Regional glucose metabolism, presynaptic dopamine transporters	2 ChAc (monozygotic twins)	Bilateral hypometabolism in C/Pu, reduced ^123^-I-FP-CIT binding in right hemisphere of one twin (corresponds to more severe left chorea), normal binding in other twin
	2010 Selcuk et al. [20]	PET	[^18^F]FDG	Regional glucose metabolism	1 ChAc	No FDG uptake in C/Pu
	2015 Cui et al. [19]	PET	[^18^F]FDG	Regional glucose metabolism	1 ChAc	Decreased in bilateral C/Pu
McLeod syndrome	2001 Jung et al. [27]	PET	[^18^F]FDG	Regional glucose metabolism	5 affected males, 2 female mutation carriers, 2 healthy males (all members of a single family)	Reduced regional glucose metabolism in C/Pu correlated with disease duration
	2001 Oechsner et al. [28]	PET	[^18^F]FDG	Regional glucose metabolism	2 affected males (unrelated), 7 HC	Reduced in C in both patients and in Pu only in one patient with chorea
	2012 Miranda et al. [53]	SPECT	^99^Tc-TRODAT-1	Dopamine transporter binding	1 McLeod syndrome	Reduced in Pu
SCA17	2005 Minnerop et al. [30]	PET, SPECT	[^18^F]FDG, ^123^-I-FP-CIT	Regional glucose metabolism, presynaptic dopamine transporters	2 SCA17	Reduced glucose metabolism in Pu in both, and in C, cerebellum, inferior and superior parietal cortex in one; reduced presynaptic dopamine transporters in C/Pu in both
	2012 Brockmann et al. [29]	PET	[^18^F]FDG, [^11^C]raclopride, [11C]-D-threo-methylphenidate	Regional glucose metabolism, D2 binding, dopamine transporters	9 SCA17 (5 symptomatic, 4 asymptomatic) of 3 unrelated families	Reduced glucose metabolism in C/Pu, cuneus, cingulum, and parietal lobe; reduced D2 levels and dopamine transporter levels in C/Pu
BHC	1986 Suchowersky et al. [31]	PET	[^18^F]FDG	Regional glucose metabolism	3 BHC, 10 HD, 7 HC	Reduced glucose metabolism in C in both BHC and HD
	2013 Konishi et al. [32]	PET	[^11^C]CFT, [^11^C]raclopride	Dopamine presynaptic transporter binding, D2 binding	2 related BHC patients with a mutation in NKX2.1 gene	Reduced [^11^C]raclopride binding and normal [^11^C]CFT binding in C/Pu
Sydenham chorea	Goldman et al. 1993 [36]	PET	[^18^F]FDG	Regional glucose metabolism	1 SC	Increased in contralateral C/Pu during chorea, return to normal levels after resolution
	1993 Heye et al. [58]	SPECT	^99m^Tc-HMPAO	Regional cerebral blood flow	1 SC	Hypoperfusion of left BG 5 days after onset of chorea
	1993 Weindl et al. [35]	PET	[^18^F]FDG	Regional glucose metabolism	2 SC	Increased in C and lentiform nucleus, return to normal levels after resolution of chorea in 1 patient
	1999 Lee et al. [59]	SPECT	^99m^Tc-ECD	Regional cerebral blood flow	1 SC	Increased perfusion of BG and thalamus acutely; normal after symptom resolution
	2002 Barsottini et al. [57]	SPECT	^99m^Tc-HMPAO	Regional cerebral blood flow	10 SC	Increased in BG in 6/10 (scanned closer to symptom onset) compared to 4/10 who exhibited normal perfusion
	2004 Demiroren et al. [60]	SPECT	^99m^Tc-HMPAO	Regional cerebral blood flow	17 SC (SPECT performed in all patients in acute phase and 6 had repeat scan after resolution of chorea)	Hyperperfusion of BG and thalamus seen in 16/17 patients in the acute phase, perfusion in the recovery phase similar to controls
	2005 Aron [38]	PET	[^18^F]FDG	Regional glucose metabolism	2 SC	Increased C/Pu acutely; normal after symptom resolution on repeat imaging in 1 patient
	2011 Paghera et al. [37]	PET	[^18^F]FDG	Regional glucose metabolism	1 SC	Increased glucose metabolism in C/Pu during acute phase with return to baseline after resolution
	2014 Beato et al. [61]	SPECT	^99m^Tc-ethyl cysteinate dimer	Regional cerebral blood flow	12 women with SC in remission, 18 HC	Hyperperfusion in left Pu in patients with SC in remission
Polycythemia vera	2008 Kim et al. [56]	SPECT	^99m^Tc-HMPAO	Regional cerebral blood flow	1 polycythemia vera with acute onset chorea	No change acutely or after symptom resolution
	2011 Huang et al. [39]	PET, SPECT	[^18^F]FDG, ^99^Tc-TRODAT	Regional glucose metabolism, dopamine transporter levels	1 polycythemia vera with left > right chorea	Increased [^18^F]FDG uptake in right dorsolateral prefrontal cortex, left insular cortex and increased ^99^Tc-TRODAT in the right C during acute chorea; return to normal after symptom resolution
Primary antiphospholipid syndrome	1998 Sunden-Cullberg et al. [40]	PET	[^18^F]FDG	Regional glucose metabolism	1 primary APL with right hemichorea	Increased in contralateral C and lentiform acutely; normal values after symptom resolution
	2009 Nordal et al. [55]	SPECT	Does not specify	Specific tracer and imaging technique not specified	1 primary APL with hemichorea	Decreased circulation in bilateral BG and medial temporal lobes acutely; normal levels after resolution of chorea
	2010 Demonty et al. [41]	PET	[^18^F]FDG	Regional glucose metabolism	1 patient with chorea associated with APL	Increased in C/Pu acutely; normal after symptom resolution
Thyroid dysfunction	2009 Yu et al. [62]	SPECT	^99m^Tc-ECD	Regional cerebral blood flow	1 patient with acute chorea as initial presentation of Graves disease	Decreased in bilateral BG, thalamus and right anterior temporal cortex
	2013 Chung et al. [42]	PET	[^18^F]FDG	Regional glucose metabolism	1 patient with chorea associated with hyperthyroidism	Increased in BG
Hyperglycemia	2007 Nguyen [43]	PET	[^18^F]FDG	Regional glucose metabolism	1 hemiballism-hemichorea associated with hyperglycemia (patient developed right chorea several weeks after nonketotic hyperosmolar coma)	Decreased in contralateral C and lentiform nucleus, increased in contralateral motor cortex
	2012 Hashimoto et al. [44]	PET	[^18^F]FDG	Regional glucose metabolism	2 diabetic patients with sudden onset hemichorea due to non-ketotic hyperglycemia	No change in C/Pu acutely; reduced in C/Pu, GP after resolution of chorea
	2014 Tan et al. [45]	PET	[^18^F]FDG	Regional glucose metabolism	2 diabetic patients with sudden onset hemichorea in setting of hyperglycemia with positive urine ketones	Increased in contralateral BG in one patient, decreased in contralateral BG in other patient at 55 days from symptom onset
	2011 Belcastro et al. [54]	SPECT	^123^-I-FP-CIT	Presynaptic dopamine transporters	1 patient with hemichorea-hemiballismus due to hyperglycemia	Reduced in contralateral Pu
DRPLA, HD, ChAc, progressive chorea and dementia of unknown etiology (HD negative), vascular hemichorea	1987 Hosokawa et al. [34]	PET	[^18^F]FDG	Regional glucose metabolism	5 patients with chorea of differing etiologies (DRPLA, HD, ChAc, progressive chorea and dementia of unknown etiology (HD negative), vascular hemichorea)	Reduced in C/Pu in all patients regardless of etiology (hypometabolism seen only on contralateral side in patient with hemichorea)
Acute stroke, non-ketotic hyperglycemia, SLE	2002 Kim et al. [63]	SPECT	^99m^Tc-HMPAO	Regional cerebral blood flow	6 patients with acute onset hemichorea (4 acute stroke, 1 non-ketotic hyperglycemia, 1 SLE	Decreased in contralateral BG; increased in thalamus

Key studies and case reports using PET and SPECT imaging in chorea of non-HD etiologies and their main findings are summarized

*Abbreviations*: *APL* antiphospholipid syndrome, *BG* basal ganglia, *BHC* benign hereditary chorea, *C* caudate, *[* ^*11*^ *C]CFT* 11-carbon-2 carbomethoxy-3-(4-[18F]-fluorophenyl)tropane, *ChAc* chorea-acanthocytosis, *DRPLA* dentatorubropallidoluysian atrophy, *[* ^*18*^ *F]FDG* ^18^F-fludeoxyglucose, *GP* Globus pallidus, *HC* healthy controls, *[* ^*123*^ *I]-FP-CIT* ^123^I-2B-carbomethoxy-3B-(4-iodophenyl)-N-(3-fluoro-propyl) nortropane), *PD* Parkinson’s Disease, *PET* Positron emission tomography, *Pu* putamen, *SCA17* spinocerebellar ataxia 17, *SLE* systemic lupus erythematosus, *SPECT* Single Photon Emission Computed Tomography, *SC* Sydenham chorea, ^*99m*^ *Tc-ECD* ^99m^Tc –ethyl cysteinate dimer, ^*99m*^ *TC-HMPAO* 99mTc-hexamethylpropyleneamineoximine, *[* ^*99m*^ *Tc]TRODAT* (99m)Tc-[2[[2-[[[3-(4-chlorophenyl)-8-methyl-8-azabicyclo[3,2,1]-oct-2-yl]-methyl](2-mercaptoeythl)amino]ethyl]amino]ethane-thiolato(3-)N2,N2’, S2,S2]oxo-[1R-exo-exo)])

In McLeod syndrome, an X-linked recessive neuroacanthocytosis syndrome, reduced striatal FDG uptake was reported in affected males [27, 28] and female mutation carriers [27]. As with HD, these observations likely correlate with neuronal loss in the caudate nucleus and putamen.

In spinocerebellar ataxia 17 (SCA17), a condition that may present with an HD-like phenotype, FDG-PET scans appeared similar to those seen in HD. A small study of symptomatic patients (*n* = 5) with SCA17 and presymptomatic SCA17 mutation carriers (*n* = 4), demonstrated decreased glucose metabolism in the caudate, putamen, cuneus, cingulum, and parietal lobe in all patients (the cerebellum was manually excluded from region of interest analysis in this study) [29]. Another small study of two patients with SCA 17, in whom chorea was not present, similarly found significantly reduced glucose metabolism in the putamen, with one patient also having reduced ^18^F-FDG uptake in the caudate nucleus, cerebellum, and the inferior and superior parietal cortices [30].

Similarly to HD, SCA 17, and ChAc, a relative reduction in glucose metabolism was also identified in the caudate in three patients with benign hereditary chorea (BHC) [31]. Additionally, a report of two related patients with BHC with an *NKX2.1* mutation demonstrated reduced relative [^11^C]-raclopride binding in the striatum with normal relative binding of 11-carbon-2 carbomethoxy-3-(4-[^18^F]-fluorophenyl)tropane ([^11^C]-CFT), a radioligand used to evaluate presynaptic dopamine transporter function [32]. This is rather surprising, as this disorder is not characterized by neurodegeneration affecting the neurons that bear dopaminergic receptors, but only of various interneurons [33]. However, the nature of dopaminergic dysfunction in BHC is as yet unclear, and the changes may reflect aberrant dopaminergic function rather than neuronal loss.

Several other conditions with chorea have also been associated with striatal hypometabolism. ^18^F-FDG-PET imaging in a patient with nonprogressive hemichorea of the shoulder, arm and chest for over 30 years, suspected to be vascular in etiology, revealed hypometabolism in the contralateral striatum [34]. Striatal glucose hypometabolism was also reported in a patient with dentatorubropallidoluysian atrophy (DRPLA) with chorea [34].

#### PET in cases of chorea from reversible etiologies

In studies of patients with chorea due to non-degenerative causes, metabolic studies tend to show increased striatal metabolism. Case reports in Sydenham chorea have demonstrated increased striatal ^18^F-FDG uptake in the striatum during the active phase of the illness with subsequent decrease or return to normal values after complete resolution of symptoms [35–38]. Similar transient hypermetabolism was demonstrated in a patient with primarily left hemi-chorea attributed to polycythemia vera, in whom there was significantly increased ^18^F-FDG uptake in the right dorsolateral prefrontal cortex and left insular cortex, with normal brain metabolism on a repeat PET scan after consecutive phlebotomy and resolution of chorea [39].

In one patient with chorea due to primary antiphospholipid syndrome, there was increased glucose metabolism in the caudate and lentiform nuclei on the side contralateral to the side of the predominant chorea, with metabolism returning to normal after treatment (with methylprednisolone) [40]. Similarly, in a more recent case of chorea (initially left-sided, but progressing to bilateral chorea) associated with anti-phospholipid antibodies, an ^18^F-FDG-PET during the acute phase showed increased bilateral striatal metabolism. A repeat scan after treatment with methylprednisolone and acetylsalicylic acid, and subsequent resolution of chorea, demonstrated normal striatal metabolism [41].

A case report of a patient with generalized chorea and hyperthyroidism demonstrated hypermetabolism in the bilateral basal ganglia on ^18^F-FDG-PET [42].

In hemichorea-hemiballism secondary to nonketotic hyperglycemia there was decreased ^18^F-FDG uptake in the contralateral caudate and lentiform nuclei, as well as increased ^18^F-FDG uptake in the contralateral motor cortex [43]. However, there was conflicting evidence from two patients with hemichorea-hemiballism of the same etiology, who exhibited normal glucose metabolism in the contralateral striatum during the acute period of chorea, which later progressed to striatal hypometabolism on follow-up ^18^F-FDG PET scan after the resolution of chorea [44]. Additional contradictory findings were presented in the case reports of two women with hemichorea-hemiballism secondary to ketotic hyperglycemia in which the FDG PET in one patient demonstrated increased glucose metabolism in the contralateral basal ganglia while the other patient exhibited a reduction in glucose metabolism in the contralateral basal ganglia. The difference in these findings and can be explained by the fact that the FDG PET scan in the patient with increased glucose metabolism was performed 9 days after onset of involuntary movements, while the scan in the patient with a reduction in glucose metabolism was performed 55 days after symptom onset (near the time of resolution of symptoms) [45].

#### Summary

^18^F-FDG PET studies of patients with neurodegenerative disorders with chorea, both symptomatic and presymptomatic, specifically HD, SCA17, DRPLA, McLeod syndrome, and ChAc, demonstrate progressive glucose hypometabolism in the striatum in addition to reduced striatal D1 and D2 receptor binding. These observations likely correlate with neuronal loss. The exception to this is benign hereditary chorea, in which there is loss only of various interneurons, yet apparently decreased striatal metabolism.

In contrast, despite the similar clinical phenomenology of chorea, striatal hypermetabolism was found in patients with hyperthyroidism, polycythemia vera, and Sydenham’s chorea. Striatal hypermetabolism tended to be seen in cases of chorea of transient etiologies in contrast to neurodegenerative etiologies of chorea, which suggests different pathophysiological mechanisms in transient vs. chronic/progressive etiologies of chorea. It is possible that the hypermetabolism seen in transient etiologies of chorea is not a manifestation of the cause of the chorea itself, but rather the result of compensatory changes that occur in the striatum and lead to the eventual resolution of chorea. Another explanation could be that this hypermetabolism reflects increased activity of the afferent corticostriatal pathway, and that this input results in hypoactivity of the indirect pathway, resulting in chorea.

Additionally, as seen in HD and other non-reversible etiologies of chorea, striatal hypometabolism and decreased labeling of D1 (post-synaptic) and D2 (pre- and post-synaptic) receptors may be important in the neuronal dysfunction that leads to the pathogenesis of chorea. Despite predictions of the basal ganglia model that chorea is primarily the consequence of degeneration of D2 receptor-bearing indirect pathway neurons, both receptor types appear to be affected.

## Single-photon emission computed tomography (SPECT)

### Neurotransmitter-related SPECT studies in HD

Although the detection techniques between SPECT and PET differ, SPECT is similar to PET in that it is a type of molecular imaging which requires the use of a molecular probe which is labeled with a radionucleotide. This results in the emission of single high-energy X-ray photons which can be detected by SPECT imaging [46]. ^123^I-2B-carbomethoxy-3B-(4-iodophenyl)-N-(3-fluoro-propyl) nortropane) ([^123^I]-FP-CIT) SPECT (DaTSCAN) is used to indicate the distribution of presynaptic dopamine transporters. In a study of twelve symptomatic HD patients of varying clinical severities, [^123^I]-FP-CIT SPECT showed reduced radioligand uptake in the putamen in eight patients and also reduced uptake in the caudate in one patient [47] (see Table 1). Another study using ^123^I-FP-CIT SPECT in HD in twelve clinically diagnosed and genetically confirmed patients with HD found a significant decrease in mean striatal, caudate, and putaminal FP-CIT uptake when compared to healthy controls. This finding was independent of striatal atrophy. While this study found no correlation between clinical and neuropsychological features/severity and degree of decrease in FP-CIT uptake [48], a more recent study of four HD patients did suggest a possible correlation between ^123^I-FP-CIT SPECT findings and UHDRS scores. In this study, three of the four patients demonstrated a decrease in ^123^I-FP-CIT in the caudate and putamen on 2-year follow-up imaging which correlated with increases of UHDRS scores while one patient showed no significant changes in striatal uptake or UHDRS scores [49]. These studies suggest that ^123^I-FP-CIT SPECT data reveals a progressive decrease in presynaptic dopaminergic dysfunction in HD and may correlate with rate of clinical progression.

SPECT studies have also demonstrated post-synaptic nigrostriatal dysfunction in HD. A study using (^99^m)Tc-[2[[2-[[[3-(4-chlorophenyl)-8-methyl-8-azabicyclo[1–3]-oct-2-yl]-methyl](2-mercaptoethyl)amino]ethyl]amino]ethane-thiolato(3-)N2,N2’, S2,S2]oxo-[1R-exo-exo)]) ([^99m^Tc]TRODAT-1) and [^123^I]Iodobenzamide ([^123^I]IBZM) SPECT in three siblings with genetically confirmed HD revealed reduced [^99m^Tc]TRODAT-1 and [^123^I]IBZM uptake compared to healthy controls indicating reduced striatal DAT and D2 receptor binding potentials respectively in HD patients, and reduction in D2 receptor binding potentials showed a correlation with functional status [50]. Another SPECT study using the radioligand [^123^I]epideride, which has a high affinity for the D2 receptor, demonstrated significantly reduced [^123^I]epideride uptake in patients with moderate or advanced stages of HD, while no changes in uptake were identified in pre-symptomatic and early HD patients [51]. This might be explained by compensatory upregulation in early stage disease, despite neuronal loss. These studies further support alterations in striatal DAT and D2 receptor binding in HD, and certain radioligands such as [^123^I]epideride demonstrate that some of these changes may not occur until later in the clinical disease course. Further knowledge of the timing and exact changes on striatal dopamine receptors could have important implications in the development of successful treatment options.

### SPECT to evaluate cerebral blood flow in HD

^99m^Technetium exametazime or ^99m^Tc bicisate SPECT can be used to examine alterations in relative cerebral perfusion. These radionucleotides were used in a group of manifest HD and pre-HD patients. A majority (88%) of the manifest HD patients showed hypoperfusion in the caudate bilaterally while only 10% of HD patients had normal SPECT findings [52]. Similar results of symmetric caudate hyperperfusion were identified in five out of twelve pre-symptomatic-HD patients while the other seven pre-symptomatic patients had normal perfusion in the caudate [52]. Similar to PET studies in HD, these findings suggest progressive neuronal dysfunction in the striatum in HD patients.

### Neurotransmitter-related SPECT in chorea of other etiologies

While studies employing the use of SPECT imaging are more numerous in HD, SPECT techniques have been used in some studies of non-HD causes of chorea (see Table 2). [^123^I]-2β-carbomethoxy-3β-(4-iodophenyl)-*N*-(3-fluoropropyl)nortropane (^123^I-FP-CIT) SPECT scans performed on a set of monozygotic twins with ChAc demonstrated reduced binding to striatal presynaptic dopamine transporters in the right hemisphere in one of the patients, which correlated to more severe left sided hyperkinetic movements in the patient; however there was no reduction in striatal dopamine transporter binding in the other twin [25]. Additionally, a case report of a man with McLeod syndrome with generalized chorea reported a decrease in dopamine transporter binding in the putamen on ^99m^Tc-TRODAT-1 SPECT scan compared to controls [53].

In a recent case report, a woman with polycythemia vera with acute onset of left hemichorea, there was reduced dopamine transporter uptake on ^99m^Tc-TRODAT-1 SPECT in the bilateral basal ganglia. Follow-up scan 10 months later, after serial phlebotomies and resolution of chorea, revealed increased TRODAT-1 uptake and were more symmetric compared to previous images [39]. Further evidence of reduced presynaptic dopamine dysfunction is described in a case report of a man with sudden onset of left hemichorea-hemiballismus due to non-ketotic hyperglycemia in which [^123^I]FP-CIT SPECT showed reduced uptake in the right putamen which correlated to the side of involuntary movements [54].

The use of SPECT in ChAc demonstrates decreased presynaptic dopamine transporter binding in the striatum. Similar findings of reduced dopamine transporter uptake were identified in the acute phase of chorea due to polycythemia vera which returned to normal after the resolution of chorea. This suggests that reduced striatal presynaptic dopamine transporter binding may serve as a biomarker for the pathogenesis of chorea independent of the etiology, however, these changes are reversible in transient causes of chorea while they are permanent in chronic/progressive causes of chorea. Given these data, we speculate that D2 receptors may be reduced in the striatum in all types of chorea, however it is possible that presynaptic dopamine transporters are later upregulated as part of the basal ganglia feedback loop in chorea of transient etiologies, thereby serving as a compensatory mechanism, which can eventually lead to resolution of the chorea.

### Perfusion SPECT in chorea of other etiologies

In a case report of a 12 year old girl with chorea of the extremities, neck, face, and tongue due to primary antiphospholipid syndrome, an initial brain SPECT showed regions of decreased perfusion in the basal ganglia and temporal lobes while perfusion returned to normal on repeat SPECT one month later [55]. However, no difference in cerebral blood flow in the acute phase of chorea compared to 6 month follow-up imaging after resolution of chorea was demonstrated in a man with generalized chorea secondary to polycythemia vera who was scanned using 99mTc-hexamethylpropyleneamineoximine (^99m^Tc-HMPAO) [56].

A study of ten patients with Sydenham chorea demonstrated hyperperfusion of the basal ganglia on ^99m^Tc HMPAO-SPECT in six of the patients, while the other four had normal SPECT. Of note, imaging was performed a mean of 49 days from symptom onset in the patients who exhibited hyperperfusion, while scans were performed after 85 days in the patients with normal SPECT findings, although this difference was not statistically significant [57]. In a case report of an 18-year-old man with bilateral Sydenham chorea, a ^99m^Tc HMPAO-SPECT during the first week of symptom onset showed hypoperfusion in the left basal ganglia [58]. In another case report in a young girl with Sydenham chorea, serial ^99m^Tc –ethyl cysteinate dimer (^99m^Tc-ECD) cerebral perfusion SPECT imaging showed increased cerebral perfusion in the striatum and thalamus during the period of active chorea, with levels similar to baseline on repeat scan after the resolution of symptoms [59]. In study of seventeen patients with Sydenham chorea, ^99m^Tc HMPAO-SPECT performed 0 to 3 weeks from symptom onset demonstrated hyperperfusion in the basal ganglia and thalamus in 94.1% of patients and was normal in one patient. Follow-up ^99m^T HMPAO-SPECT performed 6–12 months later showed reduction in perfusion compared to the acute phase and perfusion of the basal ganglia and thalamus was similar to controls [60]. Although the data in Sydenham chorea is somewhat inconsistent in the acute phase because some studies indicate hyperperfusion in the basal ganglia and others demonstrate hypoperfusion, all follow-up studies after the resolution of chorea demonstrate normal perfusion. The exception to this is one study in which SPECT scans performed after the resolution of chorea in twelve women with Sydenham chorea showed hyperperfusion in the left putamen compared to controls (of note, half of these cases had recurrent episodes of chorea) [61].

In a case report of a 17-year-old girl with acute onset chorea as her presentation of Grave’s disease, a ^99m^Tc-ECD SPECT scan demonstrated reduced perfusion to the right anterior temporal cortex while T1, T2, and diffusion weighted MRI images were normal [62].

In an analysis of six patients with hemichorea, four related to acute stroke, one with non-ketotic hyperglycemia, and one with systemic lupus erythematosus, ^99m^Tc-HMPAO SPECT in all patients demonstrated decreased perfusion of the contralateral basal ganglia (though the degree of difference did not reach statistical significance) [63].

Additionally, while transiently reduced perfusion to the basal ganglia was demonstrated in SPECT studies of chorea due to primary antiphospholipid syndrome, increased perfusion to the basal ganglia was seen in the acute phase of Sydenham’s chorea in the majority of studies. Similar to results in antiphospholipid syndrome, decreased perfusion to the basal ganglia was also seen in hemichorea due to acute stroke, SLE, and non-ketotic hyperglycemia. If we assume a common neuronal mechanism for the generation of chorea, with decreased activity of the indirect pathway, we may postulate that decreased perfusion in the basal ganglia may occur in the acute phase of chorea while hyperperfusion may occur in the recovery phase, presumably as a compensatory mechanism.

## Magnetic Resonance Spectroscopy in HD

Magnetic Resonance Spectroscopy (MRS) can be used to measure alterations in cerebral metabolism. MRS evaluations have demonstrated a reduction in N-acetylaspartate (NAA), a marker of neuronal integrity, in the putamen and caudate of pre-HD and symptomatic HD patients compared to normal controls [64, 65]. Additionally, myo-inositol, a glial cell marker, was also found to be reduced in pre-HD and manifest HD, findings which correlated with UHDRS motor scores [65]. NAA values were also decreased in 12/12 pre-symptomatic HD patients on ^1^H-MRS imaging [52], MRS evaluation in early HD patients (ten pre-HD and two with motor symptoms) demonstrated lower NAA and glutamate levels in the posterior cingulate cortex compared to normal controls, with the most pronounced difference in patients in the HD group with global cognitive impairment (Montreal Cognitive assessment scores < 26) [66]. MRS studies demonstrated evidence of neuronal dysfunction in the striatum in both HD and pre-HD, the extent of which correlated with motor impairment. Similar findings were also demonstrated in the posterior cingulate cortex, the extent of which correlated to cognitive impairment.

## Functional MRI

Functional magnetic resonance imaging (fMRI) is an MRI technique that detects changes in blood flow to measure neuronal activation. Resting state fMRI (RS-fMRI) measures spontaneous changes in blood-oxygen-level dependent (BOLD) signals to provide data about spontaneous functional changes that occur while the brain is at rest.

### Resting state fMRI in HD

A RS-fMRI study, which evaluated for potential differences in functional connectivity patterns, found no differences in connectivity between pre-HD patients and controls at baseline or after 3-year follow-up [67]. Another study which employed RS-fMRI to investigate resting state networks in early symptomatic HD patients demonstrated abnormal connectivity in several resting state motor and cognition networks compared to controls [68]. Increased connectivity in the supplementary motor area to the motor resting state network and from the left middle frontal cortices within the anterior prefrontal resting state network was identified in early HD patients [68]. Both pre-manifest and early HD patients showed reduced functional connectivity in in the left middle frontal lobe, left pre-central gyrus and right post-central gyrus with the medial visual network compared to controls. However, decreased connectivity in the left parietal lobe, bilateral pre-frontal cortices, bilateral temporal lobes and the default mode network in addition to decreased connectivity between a small part of the thalamus and the executive control network were seen only in early HD patients and not in premanifest gene carriers [69].

Another RS-fMRI study showed that alterations in network connectivity vary with disease time course. In particular, reduced synchrony was seen in the sensorimotor and dorsal attention networks in pre-HD patients compared to controls, while the left frontoparietal network demonstrated regions of increased synchrony in symptomatic HD patients compared to pre-HD. Furthermore, ﻿the posterior putamen and superior parietal cortex showed reduced connectivity with the frontal executive network in symptomatic HD compared to pre-HD or controls [70]. Another study found significant changes in resting state activity patterns in the thalamus, striatum, prefrontal cortex, premotor cortex and parietal cortex, in addition to a subset of the default mode network in HD patients compared to controls. Additionally, higher UHDRS-motor scores correlated with higher network connectivity in the motor and parietal cortices [71]. This is in contrast to a previous study, which found evidence of dysfunction in the default mode network in symptomatic HD patients, with a correlation to clinical cognitive (non-motor) disturbances [72]. In a study of RS-fMRI, which measured resting state perfusion (not BOLD), pre-symptomatic HD patients were found to have reduced regional cerebral blood flow in the medial and lateral prefrontal regions and increased regional blood flow in the precuneus, while pre-HD patients closer to the time of symptom onset also showed decreased regional blood flow in the putamen and increase rCBF in the hippocampus [73]. Although the results vary between studies, overall RS-fMRI analysis demonstrates alterations of functional connectivity in HD and suggests that some changes in connectivity may be present from pre-symptomatic stages of the disease.

### Task performance f-MRI in HD

fMRI techniques can also be employed while performing specific tasks to examine functional changes within cortical brain networks. fMRI performed during a Porteus maze task demonstrated reduced activation in the caudate, and the parietal, occipital, and somatomotor cortices and increased signal in the left postcentral and middle frontal gyri in pre-HD individuals [74]. A similar study using fMRI with a serial reaction time task showed decreased activation in early and pre-HD patients in the right middle frontal, left middle occipital, left precuneus, and left middle frontal gyri [75]. While performing a Simon effect task, patients with HD demonstrated increased activation in the caudal anterior cingulate, right inferior frontal cortex, left insula, bilateral parietotemporal cortex, left dorsal premotor and right precuneus/superior parietal regions [76]. Pre-HD patients closer to time of time of clinical diagnosis (<12 years) showed significantly decreased activation within the caudate and thalamus compared to controls, while pre-HD patients ≥12 years from time of diagnosis had an intermediate degree of decreased activation in these regions during a time discrimination task. Additionally, pre-HD patients >12 years from time of diagnosis showed increased activation of the supplementary motor area and anterior cingulate gyrus [77]. While the specific regions of overactivation and underactivation vary between studies, there does appear to be a change in task-related activation, particularly in the basal ganglia in both pre-HD and manifest HD, which supports the fact that rather than simply neuronal loss there is neuronal dysfunction. Additionally, the variability in these results may be a manifestation of neuronal dysfunction with superimposed compensatory overactivation.

### fMRI in chorea of other etiologies

The majority of fMRI studies are limited to HD patients, in fact, there are very few studies using fMRI in other etiologies of chorea. In a study of seven patients with paroxysmal kinesigenic choreoathetosis (PKC), interictal brain RS-fMRI in PKC patients showed significantly increased alteration of amplitude of low frequency fluctuation in bilateral putamen and left post-central gyrus (independent of onset side) compared to control group [78]. The results in PKC are similar to those seen in pre-HD patients, who exhibited lower functional connectivity in the bilateral putamen on fMRI.

## Conclusions

While the majority of research on chorea using functional imaging techniques has been limited to HD, the available data have revealed some interesting trends in chorea syndromes. Functional imaging has suggested the presence of not only neuronal loss in many chorea syndromes, but also neuronal dysfunction. PET and SPECT findings support the presence of alterations in metabolism and presymptomatic dopaminergic dysfunction in HD, and these changes correlate with the development of clinical symptoms. Similar biomarkers have also been identified in ChAc, McLeod syndrome, SCA 17, DRPLA with chorea, and vascular hemichorea. PET imaging has also demonstrated transient hypermetabolism in chorea with hyperthyroidism, polycythemia vera, and Sydenham’s chorea. The finding of hypermetabolism in the active phase of these transient chorea syndromes suggests that this hypermetabolism could be a compensatory mechanism ultimately leading to the resolution of the chorea. This imaging finding could potentially have prognostic implications in chorea syndromes, though further evaluation of the significance of this finding is needed.

fMRI studies have shown distinct alterations in functional connectivity in HD, including changes that occur in the presymptomatic stages of disease. Based on similar trends in PET and SPECT studies, we hypothesize that functional connectivity may be altered in other chronic etiologies of chorea, such as ChAc, SCA17, and DRPLA, though more studies are needed. Further functional imaging studies may reveal common biomarkers and regions of early dysfunction in chorea syndromes, which ultimately could help to develop more effective treatments for these conditions.

Attempts to synthesize these studies into a coherent picture are limited by the heterogeneity of methodologies and timing of studies. It would be very helpful for a standard protocol to be developed which would facilitate the correlation of studies from different centers in different disease states. Another caveat of interpretation is that medications, such as dopamine-blocking or –depleting agents, could impact imaging results. Imaging studies of patients on and off these medications will be instructive in terms of understanding their potential effects upon basal ganglia circuitry and the generation of involuntary movements.

Another limitation is the spatial resolution of imaging techniques. If the other structures of the basal ganglia, specifically the two segments of the globus pallidus and the subthalamic nucleus could be distinguished, this could potentially shed light upon their respective roles in the direct and indirect pathways in health and disease.

Functional imaging technologies hold great promise in the understanding of brain functioning in a non-invasive manner, especially in the investigation of complex brain networks such as the basal ganglia. Interpretations, however, need to take into account many factors, including the fact that changes may be primary or secondary to the underlying neurological processes. Future studies have the potential to shed further light upon disease progression and the underlying anatomic substrate for the symptoms, for example, using a standardized battery of pre- and post-synaptic dopaminergic tracers in all patients presenting with chorea regardless of etiology. These scans could be performed longitudinally, possibly starting in pre-symptomatic HD gene mutation carriers, and correlated with quantitative scales of motor function, such as the UHDRS. These types of studies could also be valuable in the evaluation of potentially disease-modifying agents, for example tracking effects upon particular neuronal classes.

## Abbreviations

[^11^C]-CFT: 11-carbon-2 carbomethoxy-3-(4-[18F]-fluorophenyl)tropane
[^11^C]FLB 457: (S)-N-((1-ethyl-2-pyrrolidinyl)methyl)-5- bromo-2,3-dimethoxybenzamide
[^11^C]SCH 23390: (R)-(+)-8-Chloro-2,3,4,5-tetrahydro-3-[11C]methyl-5-phenyl-1H-3-benzazepin-7-ol
[^123^I]IBZM: [^123^I]Iodobenzamide
[^18^F]MNI-659: 92-(2-(3-(4-(2-[^18^F]fluoroethoxy)phenyl)- 7-methyl-4-oxo-3,4-dihydroquinazolin-2-yl)ethyl)-4- isopropoxyisoindoline-1,3-dione)
[^99m^Tc]TRODAT: (99m)Tc-[2[[2-[[[3-(4-chlorophenyl)-8-methyl-8-azabicyclo[1–3]-oct-2-yl]-methyl](2-mercaptoeythl)amino]ethyl]amino]ethane-thiolato(3-)N2,N2’, S2,S2]oxo-[1R-exo-exo)])
^99m^Tc-ECD: ^99m^Tc –ethyl cysteinate dimer
^99m^Tc-HMPAO: ^99m^Tc-hexamethylpropyleneamineoximine
BHC: Benign hereditary chorea
BOLD: Blood-oxygen-level dependent
ChAc: Chorea-acanthocytosis
DaTSCAN/[^123^I]-FP-CIT: ^123^I-2B-carbomethoxy-3B-(4-iodophenyl)-N-(3-fluoro-propyl) nortropane)
DRPLA: Dentatorubropallidoluysian atrophy
FDG: ^18^F-fluorodeoxyglucose
fMRI: Functional magnetic resonance imaging
HD: Huntington’s disease
MRS: Magnetic resonance spectroscopy
NAA: N-acetylaspartate
PDE10: Phosphodiesterase 10
PET: Positron emission tomography
PKC: Paroxysmal kinesigenic choreoathetosis
RS-fMRI: Resting state fMRI
SCA17: Spinocerebellar ataxia 17
SPECT: Single Photon Emission Computed Tomography
UHDRS: Unified Huntington’s Disease Rating Scale
